# Efficacy and safety of total glucosides of paeony in the treatment of recurrent aphthous ulcers: a systematic review and meta-analysis

**DOI:** 10.3389/fphar.2024.1378782

**Published:** 2024-04-19

**Authors:** Zijian Liu, Xingyun Liu, Yangping Han, Yutian Wang, Qianyun Guo, Mingxing Lu, Shufang Li, Ying Han, Hongwei Liu

**Affiliations:** ^1^ Xiamen Key Laboratory of Stomatological Disease Diagnosis and Treatment, Stomatological Hospital of Xiamen Medical College, Xiamen, China; ^2^ Department of Oral Medicine, Peking University School and Hospital of Stomatology and National Center of Stomatology and National Clinical Research Center for Oral Diseases and National Engineering Laboratory for Digital and Material Technology of Stomatology, Beijing Key Laboratory of Digital Stomatology and Research Center of Engineering and Technology for Computerized Dentistry Ministry of Health and NMPA Key Laboratory for Dental Materials, Beijing, China; ^3^ School of Stomatology Jinan University, Guangzhou, China; ^4^ Shanghai Stomatological Hospital and School of Stomatology, Fudan University, Shanghai, China

**Keywords:** recurrent aphthous ulcers, total glucosides of paeony, efficacy, safety, meta-analysis

## Abstract

**Background:** Recurrent aphthous ulcer (RAU) had high prevalence and lacked widely recognized treatment. Total glucosides of paeony (TGP) was used in the treatment of RAU in recent years. This study was to summarize the efficacy and safety of TGP in the treatment of RAU.

**Methods:** We searched eight commonly used databases for relevant studies that published before 1 November 2023. Primary outcome was visual analogue scale (VAS). Secondary outcomes included overall response rate, significant response rate, ulcer healing time, interval, number of ulcers, and serum inflammatory factors. We conducted the meta-analysis, assessed risk of bias and the confidence of the evidence, by using Stata 15.0, Review Manager 5.4, and Gradepro.

**Results:** Nine randomized controlled trials (RCTs) encompassing 883 patients with RAU were included in the final analysis. The VAS in the TGP group was lower than that in the control group (*MD* = −1.18, *95% CI* = −1.58 to −0.78, *p* < 0.001, moderate-certainty evidence), subgroup analysis suggested longer (>8 weeks) medication and observation led to a more significant reduction in pain (*p* = 0.02). Moreover, TGP had higher overall response rate (*RR* = 1.18, *95% CI* = 1.04 to 1.33, *p* = 0.008, very low-certainty evidence) and significant response rate (*RR* = 1.72, *95% CI* = 1.38 to 2.14, *p* < 0.001, very low-certainty evidence), accelerated ulcer healing (*MD* = −1.79, *95% CI* = −2.67 to −0.91, *p* < 0.001, low-certainty evidence), and extended intervals (*MD* = 23.60, *95% CI* = 14.17 to 33.03, *p* < 0.001, very low-certainty evidence). The efficacy of TGP in reducing the number of ulcers showed no significant difference compared to the control group (*MD* = −1.66, *95% CI* = −3.60 to 0.28, *p* = 0.09, low-certainty evidence). Moreover, TGP treatment was associated with a higher incidence of abdominal symptoms (*RR* = 3.27, *95% CI* = 1.62 to 6.60, *p* < 0.001).

**Conclusion:** TGP appears to hold promise as a widely-used clinical therapeutic option for treating RAU. Nevertheless, further rigorous studies of high quality are required to validate its effectiveness.

**Systematic Review Registration**: https://www.crd.york.ac.uk/PROSPERO/display_record.php?RecordID=471154, Identifier CRD42023471154

## 1 Introduction

Recurrent aphthous ulcers (RAU), with a prevalence of 5%–66% worldwide, is the most common oral mucosal disease (Saikaly et al., 2018; Prajapat et al., 2021). The pain caused by ulcers significantly impairs patients’ ability to eat, speak, and perform other daily tasks (Lu et al., 2020). Patients who experience long-term and high-frequency RAU may become emotionally unstable and lose faith in their medical care (Hariyani et al., 2020).

The treatment goals associated with RAU can be categorized into two distinct domains. Short-term objectives encompass the reduction of pain intensity and the facilitation of ulcer healing, while long-term goals focus on mitigating the frequency of ulcerative episodes and quantity of ulcers (Lau and Smith, 2022). The attainment of short-term goals is feasible through the utilization of diverse pharmacological interventions; however, an optimal treatment strategy for long-term goals remains elusive at present.

Several drugs, including glucocorticoids, thalidomide, and colchicine, have been used in the treatment of RAU. However, the clinical application of thalidomide is considerably limited due to its teratogenic effects (Zeng et al., 2020; Amare et al., 2021; Deng et al., 2022), particularly among the young population, who are commonly affected by RAU (Cui et al., 2016). Additionally, troubling symptoms such as dizziness, constipation, and rash are challenging to mitigate. Notably, glucocorticoids frequently lead to gastrointestinal adverse reactions, and patients with obesity, glaucoma, depression, and hypertension may experience varying degrees of adverse effects, even with a dosage below 10 mg/day (Yasir et al., 2023). In the case of colchicine, the treatment of RAU may lead to gastrointestinal issues, neutropenia, and abnormal liver function, with the incidence of adverse events even surpassing that of prednisolone (Pakfetrat et al., 2010).

Although a lack of vitamins, minerals, and trace elements is thought to be one of the causes of RAU (Saikaly et al., 2018), recent studies have revealed that patients do not benefit from vitamins and minerals (Shao et al., 2018). With the exception of certain anemia patients whose RAU symptoms can be alleviated by supplementing with folic acid and vitamin B_12_ (Han and Liu H., 2022; Taleb et al., 2022), using vitamins, minerals, and trace elements to treat RAU is not advised by the most recent therapy guidelines (Guo et al., 2020; Lau and Smith, 2022; Milia et al., 2022).

Botanical drugs have gained considerable attention as researchers endeavor to achieve a delicate balance between effectiveness and potential side effects (Shavakhi et al., 2022). Total glucosides of paeony (TGP) is the total glycosides extracted from the dried roots of Paeonia lactiflora Pall. [Ranunculaceae; Paeoniae Radix Alba]. It possesses immunoregulatory properties and has been widely utilized in the treatment of autoimmune diseases (Jiang et al., 2020; Gong et al., 2022). Based on the findings of several randomized controlled trial (RCT)-based meta-analyses (Luo et al., 2017; Feng et al., 2019; Zheng et al., 2019; Wang et al., 2022; Yang et al., 2023), it is suggested that combining TGP with effective therapeutic drugs can lead to more significant treatment efficacy for Sjogren’s syndrome, systemic lupus erythematosus, psoriasis, and rheumatoid arthritis compared to using these drugs alone. However, it should be noted that the quality of the RCTs included in these meta-analyses was limited. Nonetheless, two relatively high-quality randomized, double-blinded, placebo-controlled clinical trials demonstrated the effectiveness of TGP as a standalone treatment for Sjogren’s syndrome and psoriasis (Zhou et al., 2016; Yu et al., 2017).

Traditional Chinese medicine believes that Paeonia has hepatoprotective functions (Peng et al., 2023). Recent research suggests that TGP may inhibit liver fibrosis and inflammatory response associated with cirrhosis *via* the FLI1/NLRP3 axis (Zhang et al., 2022). Previous RCTs on TGP showed no significant hepatotoxicity or ocular toxicity (Feng et al., 2019). Several large-scale meta-analyses of RCTs even indicate that the combination of TGP with other drugs reduces the incidence of hepatotoxicity compared to using the other drugs alone (Luo et al., 2017; Yu et al., 2017; Huang et al., 2019).

Previous studies have suggested various potential mechanisms by which TGP may treat RAU effectively, including the regulation of inflammatory factors such as TNF-α, IL-1β, IL-6, IL-12, TGF-β, and IL-10 (Shi et al., 2014; Giannetti et al., 2018; Zhao et al., 2018); the maintenance of a balanced ratio of CD4+/CD8+ T cells (Sun et al., 2000) and Th1/Th17 cells (Kong et al., 2018); inhibition of T-cell sensitivity to inflammation (Shi et al., 2014); and reduction in the secretion of secretory immunoglobulin A (Peng et al., 2019). The commercially available TGP capsules (trade name: Pavlin, produced by Ningbo Liwah Pharmaceutical Co., Ltd., H20055058, Paeonia lactiflora Pall. 0.3 g/capsule, containing 130 mg of paeoniflorin) is extensively utilized in clinical practice, which, to some extent, helps mitigate the heterogeneity of treatments resulting from traditional Chinese medicine empirical practices. The extraction process of TGP was shown in Supplementary Table S1.

However, the existing studies predominantly consist of small-sample trials with varying research designs. In response to these limitations, we conducted a rigorous systematic review and meta-analysis according to the Preferred Reporting Items for Systematic Reviews and Meta-Analyses (PRISMA) 2020 statement (Supplementary Table S2). Our aim was to evaluate the efficacy and safety of TGP in treating RAU.

## 2 Methods

The protocol was registered in PROSPERO (https://www.crd.york.ac.uk/PROSPERO/), with the registration number CRD42023471154.

### 2.1 Eligibility criteria

The studies were screened according to the root “PICOS” principle.

#### 2.1.1 Population

Patients diagnosed with RAU by specialist doctors in oral mucosal diseases based on typical clinical manifestations and medical history (Milia et al., 2022).

#### 2.1.2 Intervention and control

Inclusion criteria:

(1) TGP capsules were used in the intervention group;
(2) The control group was treated with vitamins (and minerals), placebos, or received the exact same medication as the intervention group, excluding TGP.

Exclusion criteria:

(1) The intervention group received traditional Chinese medicine, which consisted of peony or total glucosides of paeony, along with other Chinese herbal ingredients;
(2) Except for TGP, vitamins, minerals, and placebo, the two groups used any other different medication.

#### 2.1.3 Outcome

Primary outcome was visual analogue scale (VAS), to assess the pain intensity of ulcers. Secondary outcomes included overall response rate, significant response rate, ulcer healing time, interval (ulcer-free days in the observation period), number of ulcers, and serum inflammatory factors containing tumor necrosis factor-α (TNF-α) and interleukin-2 (IL-2). The incidence of adverse reactions was measured as a safety result.

#### 2.1.4 Study design

Inclusion criteria:

(1) Randomized controlled trials, non-randomized controlled trials and cohort studies.

Exclusion criteria:

(1) Case reports, reviews, conference articles, expert consensus, animal experiments or mechanism research;
(2) Duplicate publications, and studies with incomplete or unavailable data.

### 2.2 Data search strategy

We searched PubMed, Embase, Cochrane Library, Web of Science, China National Knowledge Infrastructure (CNKI), Wanfang Database, VIP information resource integration service platform, and China Biology Medicine Disc (SinoMed), for relevant studies that were published before 1 November 2023. The keywords searched included “Stomatitis, Aphthous” and “Paeonia”. All search strategies were presented in Supplementary Table S3. The search results were not limited by language.

### 2.3 Study selection

Two investigators (LZJ and LXY) independently selected studies. All the literature retrieved was imported into Endnote 20 to eliminate duplicates. LZJ and LXY screened the remaining literature by reading the titles and abstracts, and the full texts when needed, to determine whether they met the inclusion and exclusion criteria. Divergences were settled by consulting with a third author (LWH).

### 2.4 Data extraction

Three reviewers (LZJ, LXY, and HYP) used the prespecified form to obtain data from the papers that satisfied the criteria independently. Inconsistencies were corrected under the supervision of the responsible author (LWH). The data included authors, publication year, study design, region, sample sizes, participants’ characteristics (gender, age, and course), medication duration, observation duration, interventions, outcomes (evaluation criteria and results), and adverse events. We tried to contact the original authors for clarification when we encountered unclear data.

### 2.5 Assessment of risk of bias

Two authors (LZJ and LXY) independently assessed the risk of bias of included studies using the recommended ‘Risk of bias’ tool for trials according to the Cochrane manual. This approach addresses the following seven specific domains: (1) random sequence generation, (2) allocation concealment, (3) blinding of participants and personnel, (4) blinding of outcome assessment, (5) incomplete outcome data, (6) selective reporting, and (7) Other bias. Each item was evaluated as “high risk”, “low risk” or “unclear”. All discrepancies were resolved by discussion to reach consensus between the two review authors, with a third review author (LWH) acting as an arbiter if necessary.

### 2.6 Statistical analysis

For continuous outcomes, such as VAS, ulcer healing time, interval, number of ulcers, TNF-α, and interleukin-2, we employed the weighted mean difference. Dichotomous outcomes, such as overall response rate, significant response rate, and incidence of adverse reactions, were assessed using the risk ratio (*RR*). To quantify the effects, we provided effect sizes and 95% confidence intervals (*95%CI*) for all the analytical tools. In order to conduct the meta-analysis, we perform necessary data conversions, such as merging multiple median (interquartile spacing) and converting the median (interquartile spacing) to mean ± standard deviation (Wan et al., 2014; Luo et al., 2018). Review Manager 5.4 (http://www.cochranelibrary.com/) was used to perform data analysis.

### 2.7 Heterogeneity assessment and sensitivity analysis

The statistical heterogeneity was assessed using the *I* ^*2*^ value. If the *I* ^*2*^ exceeded 50%, it signified a notable presence of heterogeneity, and a random-effects model was chosen. Otherwise, a fixed-effects model was utilized. Subgroup analyses were used to explore the sources of heterogeneity.

Sensitivity analyses were conducted using Stata 15.0 to generate graphical representations. Additionally, for results with heterogeneity, we obtained the precise changes of *I* ^*2*^ by omitting the included studies one by one. In cases where certain studies significantly influenced the stability of the outcome, a thorough evaluation of their study design and outcome was performed. If high risks of bias or clinical heterogeneity were identified, the respective study was excluded, and a new meta-analysis was conducted using the remaining studies.

### 2.8 Subgroup analysis

Subgroup analyses were conducted based on medication duration, observation duration, and specific intervention measures employed in the control group. Notably, due to inconsistencies in efficacy evaluation criteria across studies, as indicated in Supplementary Table S4, subgroup analyses were performed specifically for the outcomes of the overall response rate and significant response rate.

### 2.9 Assessment of reporting bias

We used the Egger’s test to assess reporting bias, given the limited number of studies available (Sterne et al., 2011). It is not recommended to conduct reporting bias assessment for results based on fewer than five studies.

### 2.10 Certainty assessment

Two authors (LZJ and LXY) assessed the confidence of the evidence independently, according to the Grading of Recommendations Assessment, Development and Evaluation (GRADE) approach (https://gdt.gradepro.org/). The level of evidence was evaluated and categorized as “high”, “moderate”, “low”, or “very low."

## 3 Results

### 3.1 Literature screening

**FIGURE 1:**
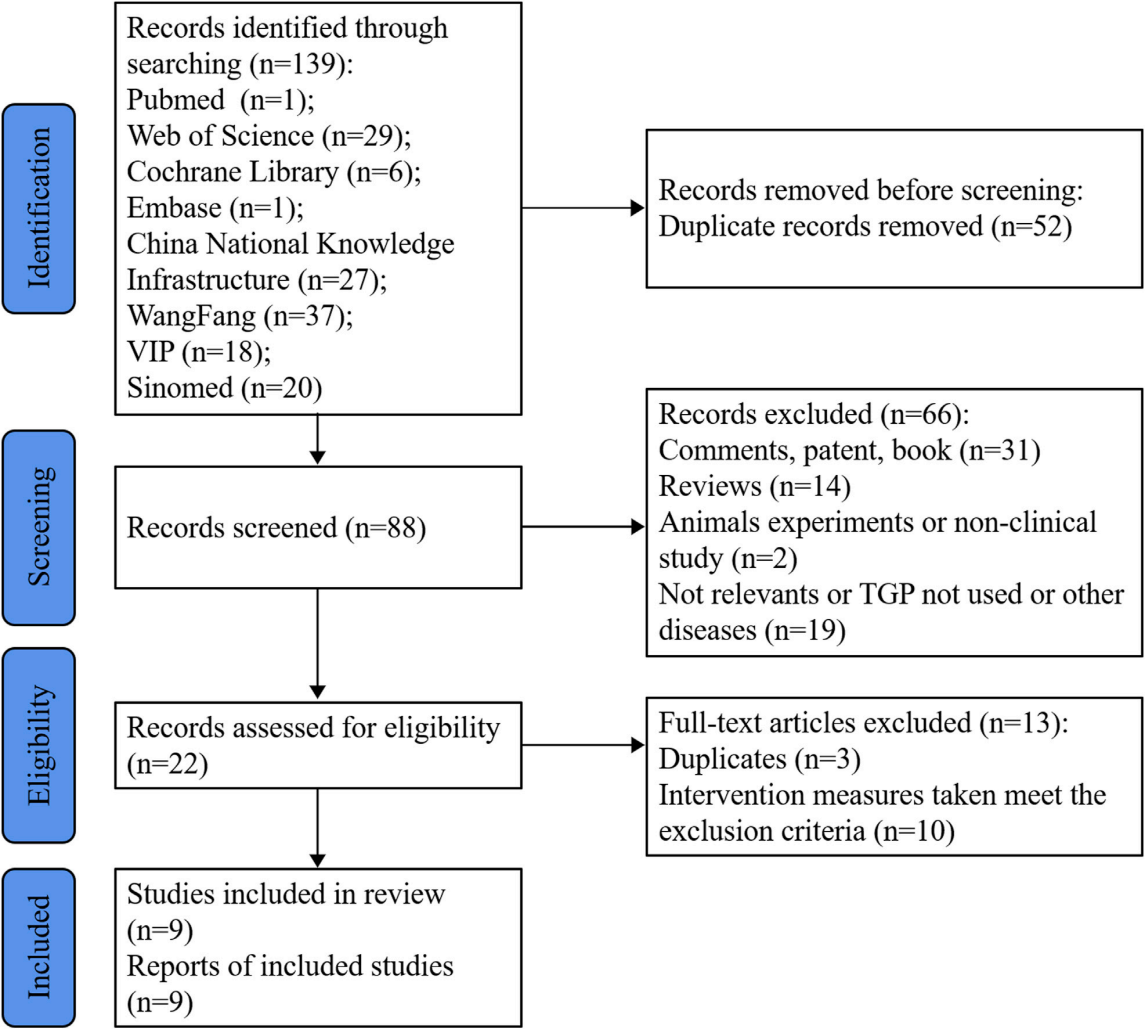
Flow diagram of the study selection process.

A comprehensive search of eight databases yielded a total of 139 records. After removing duplicates (*n* = 52), an evaluation of titles and abstracts resulted in the identification of 22 potentially eligible literature sources. Finally, nine studies (Tao, 2012; Wang et al., 2013; Su and Nong, 2014; Xu and Chen Z., 2017; Yao et al., 2017; Yan and Zhang H., 2019; Sun, 2020; Chen X. and Zhang H. L., 2021; Liu Z. et al., 2023), all of which were randomized controlled trials, were included based on a thorough examination of their full texts. The study selection process is visually depicted in Figure 1.

### 3.2 Study characteristics

All included studies were single-center RCTs conducted in eight different provinces in China. A total of 883 patients (444 in the treatment group and 439 in the control group) were enrolled. Excluding Xu et al.'s study (Xu and Chen Z., 2017), which did not report the gender and age distribution of the grouped participants, the male-to-female ratio was 328:391, with an average age range of 26.6–44.67 years. The average course in both groups ranged from 1–10 years.

The prescribed dosage of TGP for the RAU patients was 0.6 g per administration, to be taken 2–3 times daily. Only one study (Liu Z. et al., 2023) provided a comprehensive report on the actual dosage. The average daily intake of TGP and placebo during different periods ranged from 1.50 to 1.68 g, equivalent to five to six capsules per day. Several studies (Tao, 2012; Wang et al., 2013; Sun, 2020) reported cases of dose reduction in patients, but they did not specify the exact dosage and/or duration of reduced intake. The remaining studies did not mention whether the patients adhered to the prescribed medication regimen.

The medication duration exceeded 8 weeks in two studies (Xu and Chen Z., 2017; Liu Z. et al., 2023), and the observation period exceeded 8 weeks in four studies (Wang et al., 2013; Xu and Chen Z., 2017; Sun, 2020; Liu Z. et al., 2023). Both the experimental and control groups received thalidomide in two studies (Xu and Chen Z., 2017; Chen X. and Zhang H. L., 2021), one study used placebos (Liu Z. et al., 2023), and the patients in the other studies took vitamins (and minerals) as contorl (Tao, 2012; Wang et al., 2013; Su and Nong, 2014; Yao et al., 2017; Yan and Zhang H., 2019; Sun, 2020). Six studies reported VAS(Su and Nong, 2014; Xu and Chen Z., 2017; Yao et al., 2017; Yan and Zhang H., 2019; Chen X. and Zhang H. L., 2021; Liu Z. et al., 2023), while eight reported overall response rate and significant response rate (Tao, 2012; Wang et al., 2013; Su and Nong, 2014; Xu and Chen Z., 2017; Yao et al., 2017; Yan and Zhang H., 2019; Sun, 2020; Chen X. and Zhang H. L., 2021). Details were summarized in Table 1.

**TABLE 1:** Characteristics of included studies.

Authors (publication year)	Study design	Region	Sample size		Male, n (%)		Age, years		Course, years		Medication duration, week	Observation duration, week	Interventions^a^		Outcomes
			T	C	T	C	T	C	T	C			T	C	
Liu Z. et al. (2023)	RCT	Beijing, China	39	38	18 (46.2)	18 (47.4)	42.0 ± 14.5	41.2 ± 16.1	10.0 (8.0, 15.0)	9.5 (4.0, 13.0)	24	36	TGP (0.6 g tid), Kangfuxin liquid, and Tong Ren Tang Oral Ulcer Powder	Placebo, Kangfuxin liquid, and Tong Ren Tang Oral Ulcer Powder	①,④,⑤,⑧^a^
Chen X. and Zhang H. L. (2021)	RCT	Hebei, China	40	40	21 (52.50)	23 (57.50)	43.01 ± 2.82	42.67 ± 2.51	1.22 ± 0.53	1.01 ± 0.64	4	8	TGP (0.6 g bid), vitamin B, and thalidomide	Vitamin B and thalidomide	①,②,③,⑥,⑦,⑧
Sun (2020)	RCT	Shandong, China	20	19	6 (30.00)	5 (26.32)	35.3 ± 5.01	36.3 ± 4.91	NR	NR	4	12	TGP (0.6 g tid)	Vitamins with minerals tablets	②,⑧
Yan and Zhang H. (2019)	RCT	Beijing, China	56	56	26 (46.43)	27 (48.21)	44.34 ± 8.56	44.67 ± 8.49	5.91 ± 2.05	5.86 ± 2.02	1	1	TGP (0.6 g tid), iodine glycerin	Vitamin B, vitamin C, zinc, and iodine glycerin	①,②,③,⑥
Yao et al. (2017)	RCT	Zhejiang, China	60	60	28 (46.67)	31 (51.67)	29.0 ± 2.5	28.9 ± 2.5	NR	NR	8	8	TGP (0.6 g tid)	Vitamin B_2_	①,②,⑧
Xu and Chen Z. (2017)	RCT	Fujian, China	82	82	NR	NR	NR	NR	NR	NR	12	More than 1 year	TGP (0.6 g bid, 5 times per week), thalidomide, vitamin B, vitamin E, vitamin A	Thalidomide, vitamin B, vitamin E, vitamin A	①,②,③,④,⑤,⑥,⑦,⑧^b^
Su and Nong (2014)	RCT	Guangxi, China	49	49	26 (53.06)	28 (57.14)	27.3 ± 4.1	26.6 ± 5.2	0.88	0.92	8	8	TGP (0.6 g tid)	Vitamin B_2_	①,②
Wang et al. (2013)	RCT	Ningxia, China	50	50	21 (42.00)	28 (56.00)	45	48	NR	NR	8	24	TGP (0.6 g tid), Compound chlorhexidine, dexamethasone patching agent, and oral cleanser	Vitamin B_2_, Compound chlorhexidine, dexamethasone patching agent, and oral cleanser	②,④,⑤,⑧^c^
Tao (2012)	RCT	Jiangsu, China	48	45	12 (25.00)	10 (22.22)	35.6	37.2	NR	NR	4	4	TGP (0.6 g tid)	Vitamins with minerals tablets	②,⑧

T, treatment group; C, control group; RCT, randomized controlled trial; NR, not reported; TGP, total glucosides of paeony; bid, twice a day; tid, three times a day. Kangfuxin liquid, Tong Ren Tang Oral Ulcer Powder, compound chlorhexidine, dexamethasone patching agent, and oral cleanser were all used topically. ① visual analogue scale, VAS; ② overall response rate and significant response rate; ③ ulcer healing time; ④ interval; ⑤ number of ulcers; ⑥ tumor necrosis factor- α, TNF- α; ⑦ interleukin-2, IL-2; ⑧ adverse reactions.

^a^ ①, ④, and ⑤ were reported in weeks 0–4, 5–12, 13–24, and 25–36, respectively.

^b^ ①, ②, and ③ were reported in short-term observation; ②, ④, ⑤, ⑥, and ⑦ were reported in long-term observation.

^c^ ④ and ⑤ were reported every 4 weeks. Data were presented as number, the number of patients (%), mean ± standard deviation, or median (interquartile spacing).

^d^ Detailed information of the drugs, including manufacturer, batch Number, and dosage, is provided in the Supplementary Table S7.

All the included studies utilized TGP capsules, which were exclusively manufactured by Ningbo Liwah Pharmaceutical Co., Ltd. According to the “type A extract” of the ConPhyMP consensus statement (Heinrich et al., 2022), three different (orthogonal) fingerprinting methods need to be provided to verify the main ingredients of the drug. However, only one study (Liu Z. et al., 2023) showed the detection results of TGP capsule components by high-performance liquid chromatography once, which demonstrated that the content of the crucial component, paeoniflorin, in the drug was 130 mg per capsule. This amount exceeded the minimum standard set by the Chinese Pharmacopoeia 2020.

### 3.3 Risk assessment of bias

**FIGURE 2:**
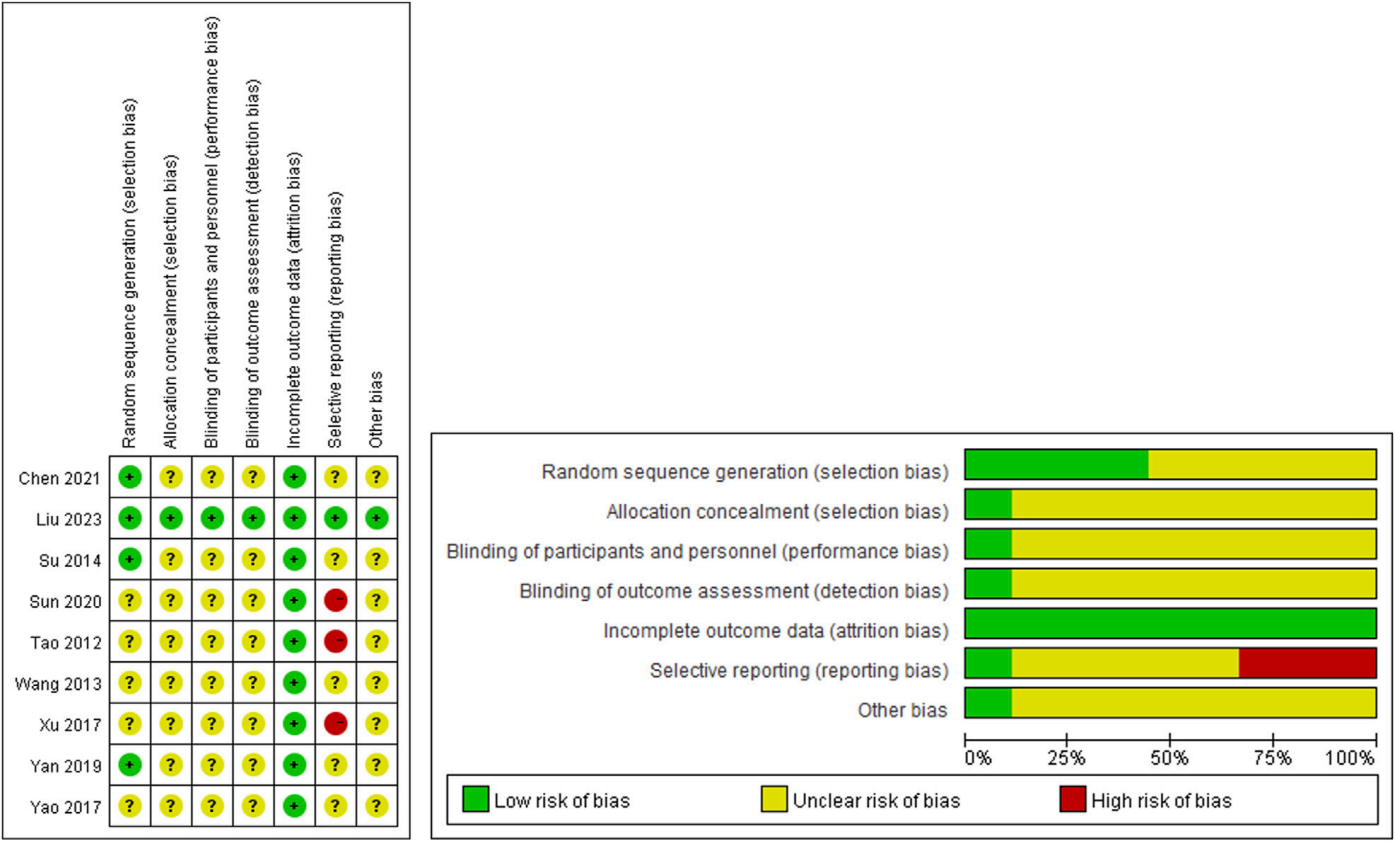
Risk of bias.

The results of the risk of bias assessment were shown in Figure 2.

Only one study (Liu Z. et al., 2023) adhered to the CONSORT 2010 Statement (http://www.consort-statement.org) and employed a double-blind randomized controlled trial design. The remaining studies were randomized controlled trials that adequately reported the main outcome measures, but did not provide information on patient withdrawal, allocation considerations, and blinding. Among these studies, only three reported utilizing the random number table method for generating random sequences (Su and Nong, 2014; Yan and Zhang H., 2019; Chen X. and Zhang H. L., 2021).

In terms of efficacy evaluation, three studies assessed efficacy using the interval and number of ulcers (Tao, 2012; Xu and Chen Z., 2017; Sun, 2020). However, these studies solely reported significant and overall response rates without specifying the interval and number of ulcers. Consequently, these studies were classified as high-risk for selective reporting due to the absence of crucial outcome indicators.

Furthermore, one study (Liu Z. et al., 2023) extensively described the use of diary cards by patients for daily recording of outcome indicators. None of the other studies provided detailed information on the recording method of outcomes, thereby raising concerns about potential recall bias in the follow-up visits.

### 3.4 Outcomes

#### 3.4.1 Primary outcome (VAS)

**FIGURE 3:**
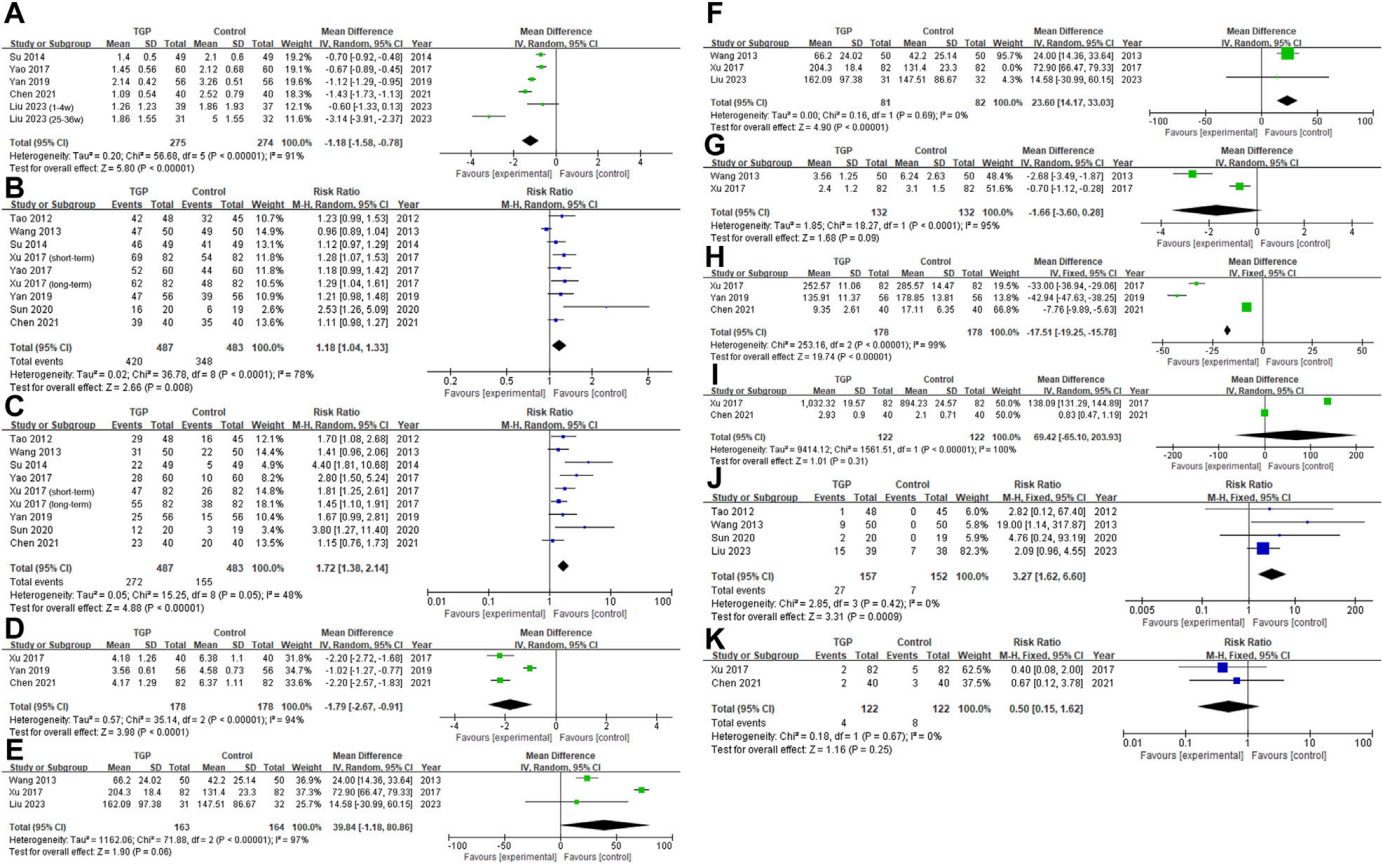
Forest plot [**(A)** VAS; **(B)** Overall response rate; **(C)** Significant response rate; **(D)** Healing time; **(E)** Interval, before deleting Xu’s study; **(F)** Interval; **(G)** Number of ulcers; **(H)** TNF-α; **(I)** IL-2; **(J)** abdominal symptoms, TGP vs. vitamin (and minerals) and placebo; **(K)** abdominal symptoms, TGP and thalidomide vs. thalidomide].

Xu reported on the VAS (Xu and Chen Z., 2017). Compared to the administration of thalidomide alone, the combination of TGP and thalidomide showed a higher VAS score. However, they concluded that the combined therapy had a better pain relief effect, which contradicts our understanding that a higher VAS indicates more severe pain. We were unable to reach the author for further clarification of the data. In order to ensure the accuracy of our research, we excluded this study in this part. Among the studies included, Liu Z. et al. (2023) reported VAS separately for the 1–4 weeks and 25–36 weeks time periods, so we included the data from these two time points independently in this study. All studies reported no intergroup differences in pre-treatment VAS. After the interventions, the VAS in the TGP groups was lower than that in the control groups (*MD* = −1.18, *95% CI* = −1.58 to −0.78, *p* < 0.001; Figure 3A), with significant statistical heterogeneity present (*I* ^*2*^ = 91%). Sensitivity analysis showed good stability (Supplementary Figure S1A), and individual study exclusion resulted in a change in *I* ^*2*^ ranging from 84% to 93% (Supplementary Table S5). Subgroup analysis suggested that the observation period and medication duration (*p* < 0.001), and treatment of the control group (*p* = 0.02) may be the sources of heterogeneity. Longer (>8 weeks) medication and observation (Liu Z. et al., 2023) resulted in a more significant reduction in pain (*MD* = −3.14, *95% CI* = −3.91 to −2.37, *p* < 0.001) compared to shorter durations (Su and Nong, 2014; Yao et al., 2017; Yan and Zhang H., 2019; Chen X. and Zhang H. L., 2021; Liu Z. et al., 2023) (*MD* = −0.93, *95% CI* = −1.23 to −0.64, *p* < 0.001). Please refer to Table 2.

**Table 2:** Subgroup analysis for outcomes.

	Number of comparisons	Participants	Results	*p*-value for overall effect	*I* ^*2*^	*p*-value for subgroup difference
**VAS**			**Mean difference (95%CI)**			
All comparisons	6	549	-1.18 [-1.58, -0.78]	<0.001	91%	
**Observation duration & Medication duration**						<0.001
≤8w	5	486	-0.93 [-1.23, -0.64]	<0.001	84%	
>8w	1	63	-3.14 [-3.91, -2.37]	<0.001	Not applicable	
**Treatment of control group**						0.02
Vitamins (and minerals)	3	330	-0.84 [-1.14, -0.53]	<0.001	85%	
Vitamins and thalidomide	1	80	-1.43 [-1.73, -1.13]	<0.001	Not applicable	
Placebo	2	139	-1.87 [-4.36, 0.62]	0.14	95%	
**Overall response rate**			**Risk Ratio (95% CI)**			
All comparisons	9	970	1.18 [1.04, 1.33]	0.008	78%	
**Medication duration**						0.43
≤8w	8	806	1.17 [1.03, 1.33]	0.02	79%	
>8w	1	164	1.29 [1.04, 1.61]	0.02	Not applicable	
**Observation duration**						0.61
≤8w	6	667	1.17 [1.09, 1.25]	<0.001	0%	
>8w	3	303	1.36 [0.77, 2.42]	0.29	94%	
**Treatment of control group**						0.82
Vitamins and thalidomide	3	408	1.20 [1.07, 1.35]	0.003	33%	
Vitamins (and minerals)	6	562	1.17 [0.98, 1.40]	0.08	83%	
**Efficacy evaluation criteria**						0.6
IN	4	396	1.29 [0.89, 1.87]	0.19	92%	
Others	5	547	1.16 [1.08, 1.25]	<0.001	0%	
**Significant response rate**			**Risk Ratio (95% CI)**			
All comparisons	9	970	1.72 [1.38, 2.14]	<0.001	48%	
**Medication duration**						0.82
≤8w	8	806	1.73 [1.47, 2.03]	<0.001	53%	
>8w	1	164	1.81 [1.25, 2.61]	0.002	Not applicable	
**Observation duration**						0.93
≤8w	6	667	1.73 [1.44, 2.08]	<0.001	58%	
>8w	3	303	1.76 [1.35, 2.28]	<0.001	38%	
**Treatment of control group**						0.04
Vitamins and thalidomide	3	408	1.49 [1.22, 1.81]	<0.001	24%	
Vitamins (and minerals)	6	562	2.04 [1.62, 2.55]	<0.001	49%	
**Efficacy evaluation criteria**						0.78
IN	4	396	1.69 [1.34, 2.13]	<0.001	6%	
Others	5	547	1.80 [1.23, 2.63]	0.002	67%	
**Healing time**			**Mean difference (95%CI)**			
All comparisons	3	356	-1.79 [-2.67, -0.91]	<0.001	94%	
**Treatment of control group**						<0.001
Vitamins and thalidomide	2	244	-2.20 [-2.50, -1.90]	<0.001	0%	
Vitamins (and minerals)	1	112	-1.02 [-1.27, -0.77]	<0.001	Not applicable	
**Interval**			**Mean difference (95%CI)**			
All comparisons	2	163	23.60 [14.17, 33.03]	<0.001	0%	
**Observation period^a^**						0.01
0-4w	2	176	0.49 [-6.84, 7.82]	0.9	81%	
5-12w	2	173	2.41 [-0.79, 5.60]	0.14	44%	
13-24w	2	165	0.98 [-6.40, 8.36]	0.79	89%	
25-36w	1	63	9.30 [5.79, 12.81]	<0.001	Not applicable	

^a^ Xu’s study was excluded from the subgroup analysis because they did not report the specific observation time. The Interval in the subgroup analysis refers to the average number of oral ulcer-free days per month during the certain observation period.

#### 3.4.2 Secondary outcome

##### 3.4.2.1 Overall response rate

Similarly, Xu (Xu and Chen Z., 2017) conducted separate reports on the overall response rate and significant response rate for short-term and long-term durations. Both sets of data were included. The TGP group demonstrated a higher overall response rate compared to the control group (*RR* = 1.18, *95% CI* = 1.04 to 1.33, *p* = 0.008, *I* ^*2*^ = 78%; Figure 3B). Sensitivity analysis indicated good stability (Supplementary Figure S1B). After excluding the study by Wang (Wang et al., 2013), the heterogeneity of the results significantly decreased (*I* ^*2*^ = 22%), as shown in Supplementary Table S5. After re-examining this study, we found no unique intervention measures or outcome evaluation criteria, and there was no high risk of bias. Therefore, we decided against excluding this study. Subgroup analysis demonstrated that neither medication duration (*p* = 0.43), observation duration (*p* = 0.61), treatment of the control group (*p* = 0.82), nor efficacy evaluation criteria (*p* = 0.60) were the sources of heterogeneity (Table 2).

##### 3.4.2.2 Significant response rate

The TGP group demonstrated a higher significant response rate (*RR* = 1.72, *95% CI* = 1.38 to 2.14, *p* < 0.001; Figure 3C). Despite the absence of significant statistical heterogeneity (*I* ^*2*^ = 48%), we opted for a random-effects model considering the varying treatment measures and efficacy evaluation criteria across studies. The sensitivity analysis confirmed the stability of the results (Supplementary Figure S1C). The difference in significant response rate between the TGP group and the control group, when the control group using both vitamin and thalidomide (Xu and Chen Z., 2017; Chen X. and Zhang H. L., 2021) (*RR* = 1.49, *95% CI* = 1.22 to 1.81, *p* < 0.001), was smaller compared to the control group using vitamins (and minerals) (Tao, 2012; Wang et al., 2013; Su and Nong, 2014; Yao et al., 2017; Yan and Zhang H., 2019; Sun, 2020) (*RR* = 2.04, *95% CI* = 1.62 to 2.55, *p* < 0.001), with a *p*-value of 0.04. See Table 2.

##### 3.4.2.3 Healing time

TGP accelerated ulcer healing (*MD* = −1.79, *95% CI* = −2.67 to −0.91, *p* < 0.001; Figure 3D), and this result was stable (Supplementary Figure S1D). The heterogeneity (*I* ^*2*^ = 94%) may be attributed to specific intervention measures. In comparison to the study where the control group received only vitamins (and minerals) (Yan and Zhang H., 2019) (*MD* = −1.02, *95% CI* = −1.27 to 0.77, *p* < 0.001), the studies involving the administration of thalidomide in both the control and TGP groups (Xu and Chen Z., 2017; Chen X. and Zhang H. L., 2021) (*MD* = −2.20, *95% CI* = −2.50 to −1.90, *p* < 0.001) demonstrated a more significant difference in the healing time of ulcers between the two groups, *p* < 0.001 (Table 2).

##### 3.4.2.4 Interval

TGP appeared to prolong the interval, but lacked statistical significance and exhibited heterogeneity (*MD* = 39.84, *95% CI* = −1.18 to 80.86, *p* = 0.06, *I* ^*2*^ = 97%; Figure 3E). Sensitivity analysis indicated that removing Xu’s study (Xu and Chen Z., 2017) could enhance outcome stability (Supplementary Figure S1E), with *I* ^*2*^ decreasing to 0% (Supplementary Table S5). Considering that Xu’s study (Xu and Chen Z., 2017) was the only one among the studies reported on intervals that utilized thalidomide, did not report the overall observation duration, and exhibited significant reporting bias, we reanalyzed the remaining studies. The results demonstrated that TGP could prolong the interval (*MD* = 23.60, *95% CI* = 14.17 to 33.03, *p* < 0.001, *I* ^*2*^ = 0%; Figure 3F). Subgroup analysis was conducted based on the following intervals: 0–4 weeks, 5–12 weeks, 13–24 weeks, and 25–36 weeks. Within the 0–24 weeks period, Wang’s study (Wang et al., 2013) showed that TGP prolonged the interval, while Liu’s study (Liu Z. et al., 2023) indicated a lack of significant difference between TGP and placebo (Supplementary Figure S2). The pooled analysis results indicated no significant prolongation compared to the control group (0–4 weeks: *p* = 0.90, 5–12 weeks: *p* = 0.14, 13–24 weeks: *p* = 0.79; Table 2). However, within the 25–36 weeks period, TGP significantly prolonged the interval (*MD* = 9.30, *95% CI* = 5.79 to 12.81, *p* < 0.001; Table 2).

##### 3.4.2.5 Number of ulcers

Three studies reported the number of ulcers. However, Liu’s study (Liu Z. et al., 2023) employed a totally different calculation method for the number of ulcers compared to the other two studies, making it impossible to convert and combine the data. The remaining two studies (Wang et al., 2013; Xu and Chen Z., 2017) demonstrated that TGP’s ability to reduce the number of ulcers did not significantly differ from the control group (*MD* = −1.66, *95% CI* = −3.60 to 0.28, *p* = 0.09, *I* ^*2*^ = 95%; Figure 3G).

Xu and Chen Z. (2017) did not report the specific observation time. Similar to the results for the interval, Wang’s study (Wang et al., 2013) indicated a reduction in the number of ulcers within 0–24 weeks (*p* < 0.01), while Liu Z. et al. (2023) showed a reduction in the number of ulcers only in the 25–26 weeks (*p* < 0.001). See Supplementary Table S6 for details.

##### 3.4.2.6 Serum inflammatory factors

TGP was found to significantly decrease serum TNF-α levels (*MD* = −17.51, *95% CI* = −19.25 to 15.78, *p* < 0.001, *I* ^*2*^ = 99%; Figure 3H), while its effect on IL-2 was not significant (*MD* = 69.42, *95% CI* = −65.10 to 203.93, *p* = 0.31, *I* ^*2*^ = 100%; Figure 3I). Although each individual study demonstrated the efficacy of TGP in reducing serum inflammatory factors, a notable disparity in the levels of serum inflammatory factors was observed between Chen’s study (Chen X. and Zhang H. L., 2021) and other studies (Xu and Chen Z., 2017; Yan and Zhang H., 2019). This dissimilarity could potentially account for the significant heterogeneity and the absence of statistical significance. However, we were unable to pinpoint a specific reason for this disparity based on the methodology employed.

#### 3.4.3 Safety outcomes

Two studies (Su and Nong, 2014; Yan and Zhang H., 2019) did not report adverse reactions, while five studies indicated that taking TGP was associated with a higher incidence of abdominal symptoms, primarily characterized by increased stool frequency, loose stools, or diarrhea. Among them, four studies (Tao, 2012; Wang et al., 2013; Sun, 2020; Liu Z. et al., 2023) reported a specific number of individuals experiencing adverse reactions, suggesting a significantly elevated likelihood of abdominal symptoms in the TGP group compared to the vitamin, minerals, and placebo (*RR* = 3.27, *95% CI* = 1.62 to 6.60, *p* < 0.001, *I* ^*2*^ = 0%; Figure 3J). The abdominal symptoms disappeared when patients discontinued or reduced the dosage of TGP.

Additionally, Wang et al. (2013) reported that among 50 patients receiving TGP, five individuals experienced symptoms of nausea and mild headache, while four individuals experienced decreased appetite. These symptoms resolved after 4–7 days without any intervention. Yao et al. (2017) reported the same adverse reactions but did not provide detailed information regarding the number of affected individuals, duration of symptoms, and persistence post-treatment cessation.

In two studies (Xu and Chen Z., 2017; Chen X. and Zhang H. L., 2021) that used thalidomide in the control group, the combined use of TGP was found to reduce the incidence of abdominal symptoms, but the intergroup difference lacked statistical significance (*RR* = 0.50, *95% CI* = 0.15 to 1.62, *p* = 0.25, *I* ^*2*^ = 0%; Figure 3K). Furthermore, TGP was also found to decrease dizziness and drowsiness caused by thalidomide.

Only one study (Liu Z. et al., 2023) reported that after 6 months of drug administration, the blood biochemical parameters of patients were examined, including alanine aminotransferase, aspartate aminotransferase, bilirubin, and albumin. The results showed no abnormalities, and there were no significant differences in the measured values compared to before the medication, indicating that TGP does not affect liver function. Other studies did not mention monitoring and evaluation of liver function.

### 3.5 Publication bias

Egger’s tests uncovered the existence of publication bias in both the overall response rate (*p* < 0.001) and the significant response rate (*p* = 0.006). There was no conclusive indication of publication bias detected for the VAS (*p* = 0.201). Egger’s linear regression test was shown in Supplementary Figure S3.

### 3.6 GRADE assessment

**FIGURE 4:**
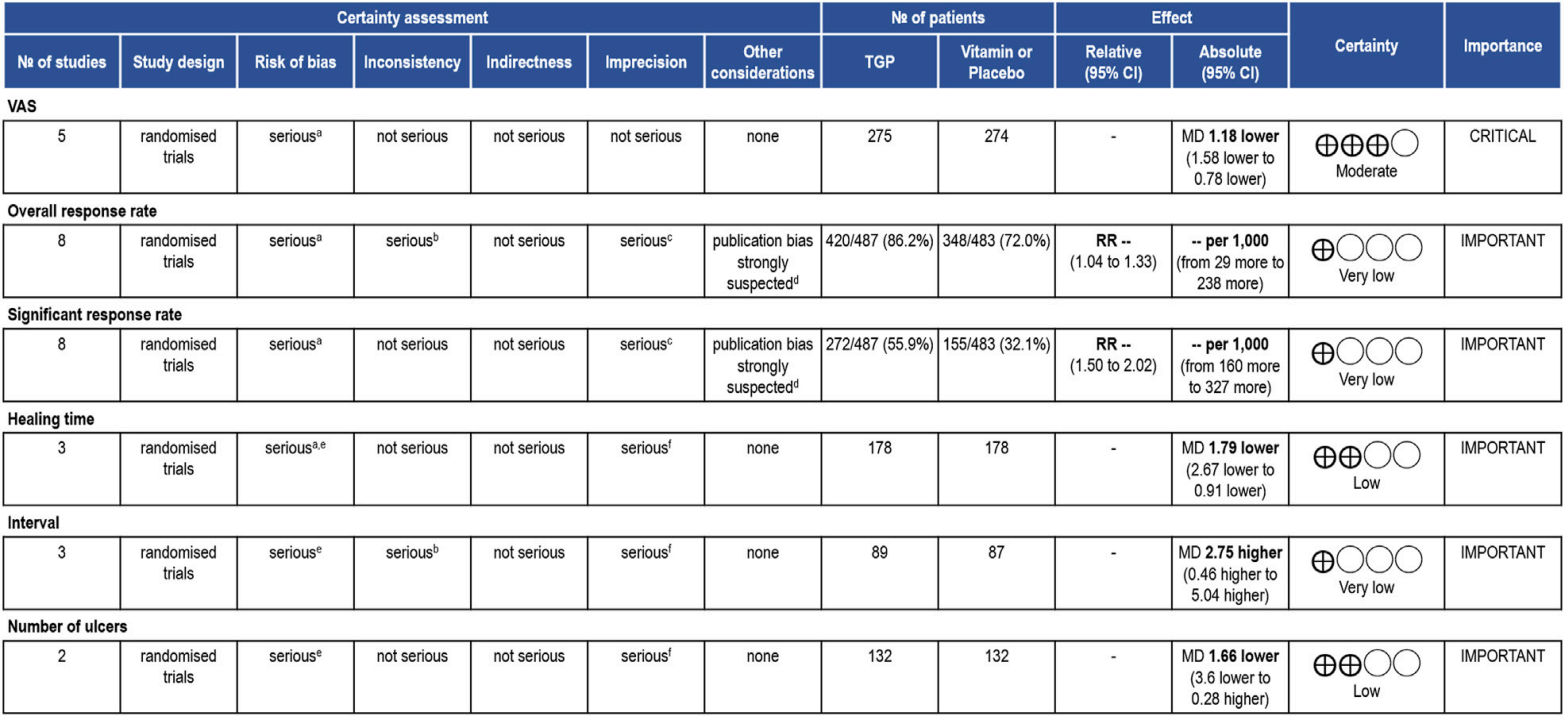
GRADE assessment. TGP compared to control treatment for RAU. *CI:* confidence interval; *MD*: mean difference; *RR*: risk ratio. Reasons for downgrading: ^a^The methods of randomization, allocation concealment and blinding were unclear. ^b^There was serious heterogeneity among the studies, and no reasonable explanation was found. ^c^The judgment method for outcome indicators was vague or did not report specific key quantitative values. ^d^Potential publication bias was detected by quantitative methods. ^e^ One of the studies had high reporting bias. ^f^ The sample size was small and the confidence interval of the outcome was wide.

We conducted a GRADE assessment only for the clinical outcomes. All the outcomes exhibited a serious risk of bias. There was significant heterogeneity among the studies for the overall response rate and interval, but no reasonable explanation could be identified. Except for the VAS, other outcomes suffered from imprecision due to vague judgment methods, the absence of specific key quantitative values in the reporting, or small sample sizes and wide confidence intervals. Egger’s tests detected publication bias in the overall response rate and significant response rate. The certainty of evidence for the VAS was rated as moderate, while for the rest of the outcomes, it was low or even very low. Figure 4 provided an overview of the evidence certainty.

## 4 Discussion

### 4.1 Summary of findings

In comparison to the utilization of vitamins (and minerals), placebos, or no alternative treatments, the administration of TGP, either alone or in combination with the same drugs used in the control group, demonstrated superior pain relief, higher response rates, and accelerated ulcer healing. However, apart from the significant response rate, all the other results mentioned above exhibit heterogeneity. Subgroup analysis revealed that TGP treatment exceeding 2 months resulted in enhanced pain relief. Furthermore, the combined use of thalidomide and TGP significantly shortened the healing time of ulcers.

Regarding the two indicators representing long-term efficacy, “interval” and “number of ulcers”. Taking into account the limitations and heterogeneity of Xu’s study, we conducted a meta-analysis after excluding it, indicating a significant prolongation of the interval with TGP, while subgroup analysis suggested a significant extension only after medication for 6 months. Three articles reported the “number of ulcers”. Similarly, it seems that a distinct decrease in the number of ulcers could be observed only after a 6-month medication of TGP. The included literature collectively demonstrated that TGP was able to lower serum inflammation levels, although the analysis results for IL-2 did not show statistical significance.

It should be noted that TGP may induce abdominal symptoms and alterations in stool characteristics, which return to normal after discontinuation. On the other hand, TGP might potentially reduce the incidence of adverse reactions associated with thalidomide, and accelerated the rate of ulcer healing facilitated by thalidomide.

Publication bias and issues such as small sample sizes diminished our confidence in these results. The GRADE assessment indicated that TGP’s effect on alleviating pain was relatively reliable, while the evidence grade for the remaining outcomes was low or even very low.

### 4.2 Strengths and limitations

We employed multiple outcomes and conducted various subgroup analyses based on specific intervention measures and durations, demonstrating the efficacy and safety of TGP in treating RAU. However, considering the various limitations, we interpret these results with caution, and further studies are warranted to validate these findings. To our knowledge, this is the first meta-analysis examining the use of TGP in the treatment of RAU.

Inevitably, several limitations should be acknowledged in this meta-analysis.

1) All the included studies were conducted in China, limiting the generalizability of the findings to other populations. These studies employed different intervention measures and durations, and reported varying outcomes, resulting in a limited number of studies included in a certain outcome and contributed to heterogeneity. This may potentially affect the scientific validity and reliability of the conclusions.
2) The administration of active ingredients by the patients remains unclear. Apart from Liu’s study (Liu Z. et al., 2023), other studies did not report the actual dosage of medication taken by the patients. On the other hand, due to the lack of fingerprinting results for drug samples, all included studies were unable to ascertain the true dosage of active ingredients in the medication consumed by the patients.
3) Only one study provided detailed descriptions of the blinding, randomization, and specific outcome measurement methods, while the remaining studies did not employ blinding and lacked detailed methodological reporting. The reporting of outcomes was not sufficiently detailed, with only three studies reporting specific indicators such as the interval and number of ulcers (Tao, 2012; Xu and Chen Z., 2017; Sun, 2020). Other studies only reported effective rates, but these rates were based on evaluations using indicators such as the interval and number of ulcers. This lead to imprecision. Ideally, the evaluation of therapeutic efficacy should be derived from daily patient records. Otherwise, relying on patient reports during follow-up visits would inevitably introduce recall bias. However, this method was only applied in the study by Liu et al. (Liu Z. et al., 2023).

Furthermore, the relatively high-quality study reported that TGP required more than 6 months of usage to achieve significant efficacy in VAS, interval, and number of ulcers, which differed from other study results, indicating substantial heterogeneity. This to some extent reduced the accuracy of the results of this meta-analysis.

4) Except for the VAS, the results of the overall response rate and significant response rate were susceptible to publication bias. Other outcomes had limited studies inclusion, making them not recommended for publication bias assessment, but this did not imply the absence of publication bias.
5) Safety evaluation did not receive sufficient attention in most studies, with a lack of detailed reporting on the duration of adverse reactions, mitigation measures, and monitoring of liver and kidney function.

Considering the aforementioned limitations, more research is needed to further validate our results. Future trials should adhere to rigorous methodology, encompassing a calculated sample size, extended follow-up period, pre-registered protocol, and implementation of a blinded method. Besides, the reporting of results should align with the guidelines provided by SPIRIT-TCM Extension 2018 (Dai et al., 2019) and CONSORT-CHM Formulas 2017 (Cheng et al., 2017).

## 5 Conclusion

This meta-analysis indicates that TGP demonstrates potential effectiveness in the treatment of RAU, particularly in alleviating pain, with no severe adverse effects observed. However, due to the significant heterogeneity and low quality of evidence, further large-scale, high-quality studies are necessary to substantiate and confirm the clinical efficacy of TGP in the RAU treatment.

## Data Availability

The original contributions presented in the study are included in the article/Supplementary Material, further inquiries can be directed to the corresponding author.
