# Supplementary material for: Efficacy and safety of total glucosides of paeony in the treatment of recurrent aphthous ulcers: a systematic review and meta-analysis
Source: Front Pharmacol. 2024 Apr 19;15:1378782. doi: 10.3389/fphar.2024.1378782 (PMC11074776; doi:10.3389/fphar.2024.1378782)
Supplement: Supplementary file 1 [file DataSheet1.pdf]

Efficacy and safety of total glucosides of paeony in the treatment of recurrent aphthous ulcers: a systematic review and meta-analysis

Supplementary Material

1 Supplementary Figures

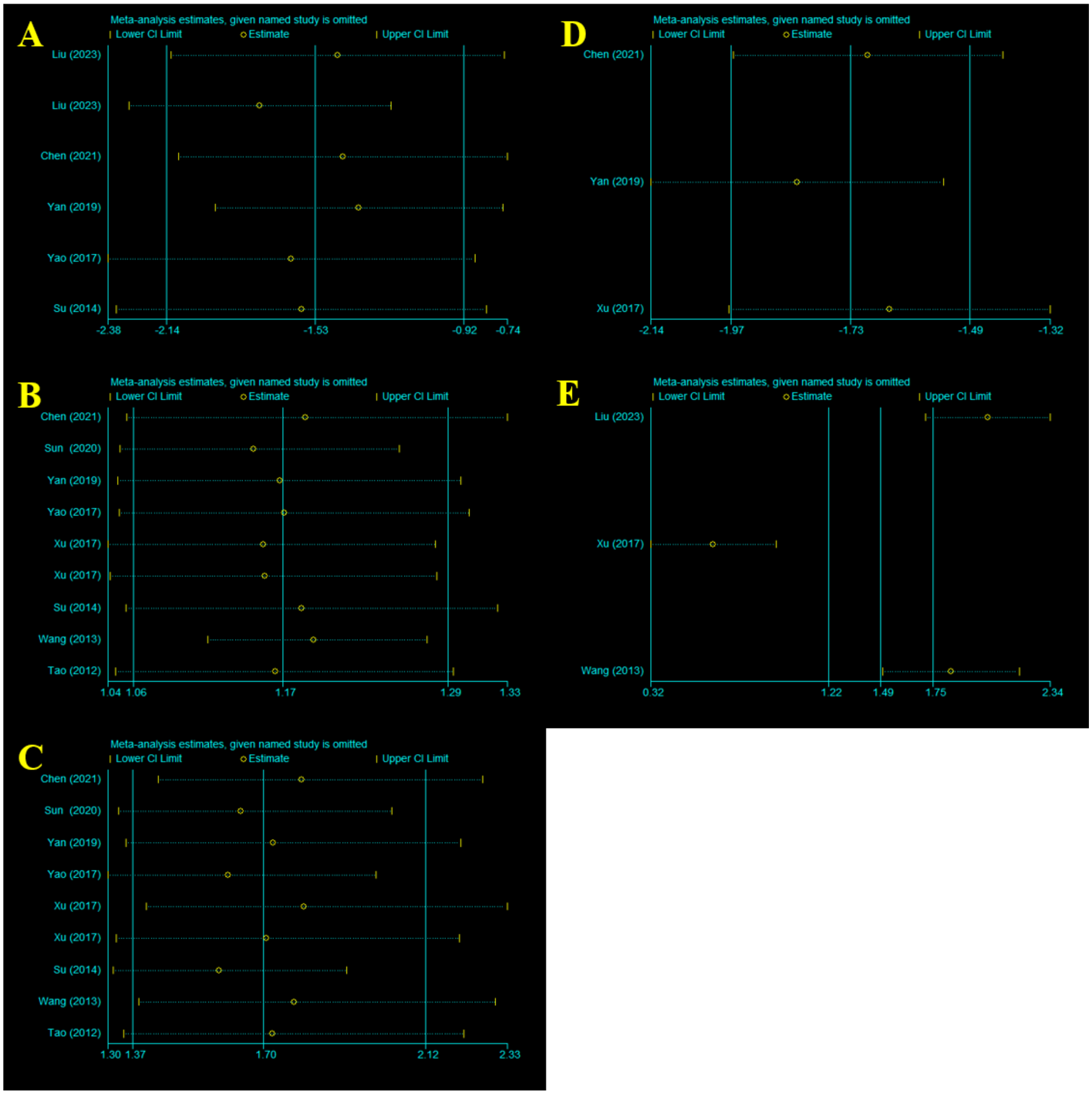

Supplementary Figure S1. Sensitivity analysis [(A) VAS; (B) Overall response rate; (C) Significant response rate; (D) Healing time; (E) Interval, before deleting Xu's study].

Study or Subgroup	TGP Mean	TGP SD	TGP Total	Control Mean	Control SD	Control Total	Weight	Mean Difference IV, Random, 95% CI	Year
**4.1.1 Interval$_{(0\text{-}4w)}$**									
Wang 2013	9.6	3.93	50	5.96	3.26	50	19.4%	3.64 [2.22, 5.06]	2013
Liu 2023	9.74	14.01	39	13.69	14.04	37	8.1%	-3.95 [-10.26, 2.36]	2023
**Subtotal (95% CI)**			**89**			**87**	**27.5%**	**0.49 [-6.84, 7.82]**	
Heterogeneity: Tau² = 23.36; Chi² = 5.29, df = 1 (P = 0.02); I² = 81%									
Test for overall effect: Z = 0.13 (P = 0.90)									
**4.1.2 Interval$_{(5\text{-}12w)}$**									
Wang 2013	10.42	5.19	50	6.94	4.89	50	18.1%	3.48 [1.50, 5.46]	2013
Liu 2023	13.9	13.64	38	13.97	6.18	35	10.9%	-0.07 [-4.87, 4.73]	2023
**Subtotal (95% CI)**			**88**			**85**	**29.0%**	**2.41 [-0.79, 5.60]**	
Heterogeneity: Tau² = 2.80; Chi² = 1.80, df = 1 (P = 0.18); I² = 44%									
Test for overall effect: Z = 1.47 (P = 0.14)									
**4.1.3 Interval$_{(13\text{-}24w)}$**									
Wang 2013	11.95	5.63	50	7.45	5.45	50	17.6%	4.50 [2.33, 6.67]	2013
Liu 2023	17.4	9.38	33	20.45	8.77	32	11.8%	-3.05 [-7.46, 1.36]	2023
**Subtotal (95% CI)**			**83**			**82**	**29.4%**	**0.98 [-6.40, 8.36]**	
Heterogeneity: Tau² = 25.35; Chi² = 9.05, df = 1 (P = 0.003); I² = 89%									
Test for overall effect: Z = 0.26 (P = 0.79)									
**4.1.4 Interval$_{(25\text{-}36w)}$**									
Liu 2023	24.13	3.11	31	14.83	9.62	32	14.0%	9.30 [5.79, 12.81]	2023
**Subtotal (95% CI)**			**31**			**32**	**14.0%**	**9.30 [5.79, 12.81]**	
Heterogeneity: Not applicable									
Test for overall effect: Z = 5.20 (P < 0.00001)									
**Total (95% CI)**			**291**			**286**	**100.0%**	**2.75 [0.46, 5.04]**	
Heterogeneity: Tau² = 6.51; Chi² = 27.28, df = 6 (P = 0.0001); I² = 78%									
Test for overall effect: Z = 2.35 (P = 0.02)									
Test for subgroup differences: Chi² = 10.83, df = 3 (P = 0.01), I² = 72.3%									

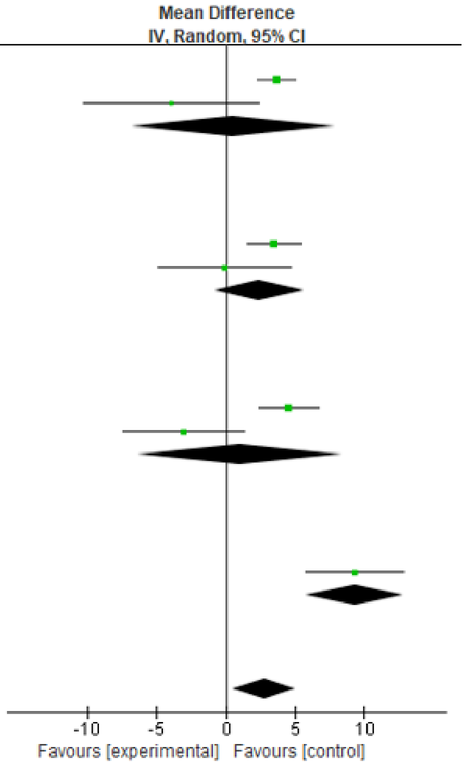

Supplementary Figure S2. Subgroup analysis of interval.

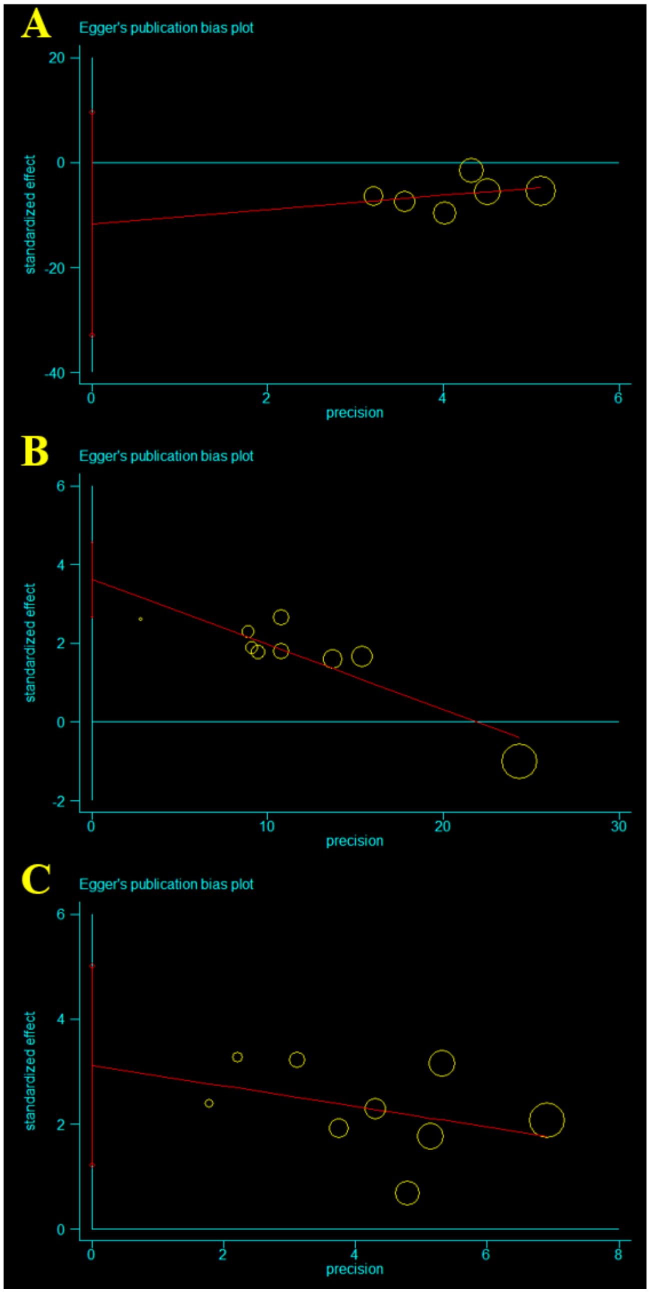

Supplementary Figure S3. Publication bias [(A) VAS; (B) Overall response rate; (C) Significant response rate].

2 Supplementary Tables

Supplementary Table S1. Extraction process of TGP.

Extraction process of TGP
100 g of sliced dried roots of Paeonia lactiflora Pall. obtained in Changchun was mixed with 400mL of a 75% ethanol-water solution and heated under the reflux system for 1.5 hours for the first and second time, and 1 hour for the third time. The solution was then filtered and concentrated to the appropriate volume, and $NaHCO_3$ solution was added to adjust the pH to 5.9-6.1. The aqueous solution was extracted with 30 mL of 80% (V/V) butanol-ethyl acetate solution three times at 50℃～65℃, with a target relative density of 1.13-1.20. The extracts were combined, concentrated under reduced pressure, and dried in vacuo to yield dry powder. Finally, a total of 6.8g of dry powder was obtained. No excipients were added. The calculated content of paeoniflorin in the drug has to exceed 104mg/0.3g, according to the standard of the Chinese Pharmacopoeia 2020.

Supplementary Table S2. PRISMA 2020 Checklist.

Section and Topic	Item #	Checklist item	Location where item is reported
**TITLE**			
Title	1	Identify the report as a systematic review.	
**ABSTRACT**			
Abstract	2	See the PRISMA 2020 for Abstracts checklist.	
**INTRODUCTION**			
Rationale	3	Describe the rationale for the review in the context of existing knowledge.	
Objectives	4	Provide an explicit statement of the objective(s) or question(s) the review addresses.	
**METHODS**			
Eligibility criteria	5	Specify the inclusion and exclusion criteria for the review and how studies were grouped for the syntheses.	
Information sources	6	Specify all databases, registers, websites, organisations, reference lists and other sources searched or consulted to identify studies. Specify the date when each source was last searched or consulted.	
Search strategy	7	Present the full search strategies for all databases, registers and websites, including any filters and limits used.	Supplementary Table S3
Selection process	8	Specify the methods used to decide whether a study met the inclusion criteria of the review, including how many reviewers screened each record and each report retrieved, whether they worked independently, and if applicable, details of automation tools used in the process.	
Data collection process	9	Specify the methods used to collect data from reports, including how many reviewers collected data from each report, whether they worked independently, any processes for obtaining or confirming data from study investigators, and if applicable, details of automation tools used in the process.	
Data items	10a	List and define all outcomes for which data were sought. Specify whether all results that were compatible with each outcome domain in each study were sought (e.g. for all measures, time points, analyses), and if not, the methods used to decide which results to collect.	
	10b	List and define all other variables for which data were sought (e.g. participant and intervention characteristics, funding sources). Describe any assumptions made about any missing or unclear information.	
Study risk of bias assessment	11	Specify the methods used to assess risk of bias in the included studies, including details of the tool(s) used, how many reviewers assessed each study and whether they worked independently, and if applicable, details of automation tools used in the process.	
Effect measures	12	Specify for each outcome the effect measure(s) (e.g. risk ratio, mean difference) used in the synthesis or presentation of results.	
Synthesis methods	13a	Describe the processes used to decide which studies were eligible for each synthesis (e.g. tabulating the study intervention characteristics and comparing against the planned groups for each synthesis (item #5)).	

Section and Topic	Item #	Checklist item	Location where item is reported
	13b	Describe any methods required to prepare the data for presentation or synthesis, such as handling of missing summary statistics, or data conversions.	
	13c	Describe any methods used to tabulate or visually display results of individual studies and syntheses.	
	13d	Describe any methods used to synthesize results and provide a rationale for the choice(s). If meta-analysis was performed, describe the model(s), method(s) to identify the presence and extent of statistical heterogeneity, and software package(s) used.	
	13e	Describe any methods used to explore possible causes of heterogeneity among study results (e.g. subgroup analysis, meta-regression).	
	13f	Describe any sensitivity analyses conducted to assess robustness of the synthesized results.	
Reporting bias assessment	14	Describe any methods used to assess risk of bias due to missing results in a synthesis (arising from reporting biases).	
Certainty assessment	15	Describe any methods used to assess certainty (or confidence) in the body of evidence for an outcome.	
**RESULTS**			
Study selection	16a	Describe the results of the search and selection process, from the number of records identified in the search to the number of studies included in the review, ideally using a flow diagram.	Figure 1
	16b	Cite studies that might appear to meet the inclusion criteria, but which were excluded, and explain why they were excluded.	Figure 1
Study characteristics	17	Cite each included study and present its characteristics.	Table 1
Risk of bias in studies	18	Present assessments of risk of bias for each included study.	Figure 2
Results of individual studies	19	For all outcomes, present, for each study: (a) summary statistics for each group (where appropriate) and (b) an effect estimate and its precision (e.g. confidence/credible interval), ideally using structured tables or plots.	Figure 3
Results of syntheses	20a	For each synthesis, briefly summarise the characteristics and risk of bias among contributing studies.	Figure 2, Figure 3, Table 1
	20b	Present results of all statistical syntheses conducted. If meta-analysis was done, present for each the summary estimate and its precision (e.g. confidence/credible interval) and measures of statistical heterogeneity. If comparing groups, describe the direction of the effect.	Figure 3
	20c	Present results of all investigations of possible causes of heterogeneity among study results.	Figure 3, Table 2, Supplementary Figure S2, Supplementary Table S4, Supplementary Table S6
	20d	Present results of all sensitivity analyses conducted to assess the robustness of the synthesized results.	Supplementary Figure S1, Supplementary Table S5
Reporting biases	21	Present assessments of risk of bias due to missing results (arising from reporting biases) for each synthesis assessed.	Supplementary Figure S3

Section and Topic	Item #	Checklist item	Location where item is reported
Certainty of evidence	22	Present assessments of certainty (or confidence) in the body of evidence for each outcome assessed.	Figure 4
**DISCUSSION**			
Discussion	23a	Provide a general interpretation of the results in the context of other evidence.	
	23b	Discuss any limitations of the evidence included in the review.	
	23c	Discuss any limitations of the review processes used.	
	23d	Discuss implications of the results for practice, policy, and future research.	
**OTHER INFORMATION**			
Registration and protocol	24a	Provide registration information for the review, including register name and registration number, or state that the review was not registered.	
	24b	Indicate where the review protocol can be accessed, or state that a protocol was not prepared.	
	24c	Describe and explain any amendments to information provided at registration or in the protocol.	
Support	25	Describe sources of financial or non-financial support for the review, and the role of the funders or sponsors in the review.	
Competing interests	26	Declare any competing interests of review authors.	
Availability of data, code and other materials	27	Report which of the following are publicly available and where they can be found: template data collection forms; data extracted from included studies; data used for all analyses; analytic code; any other materials used in the review.	

Supplementary Table S3. The search strategies for all databases.

The search strategy for PubMed	
Number	Search terms
#1	Stomatitis, Aphthous [MeSH Terms]
#2	Aphthous Stomatitides [Title/ Abstract]
#3	Aphthous Stomatitis [Title/ Abstract]
#4	Stomatitides, Aphthous [Title/ Abstract]
#5	Ulcer, Aphthous [Title/ Abstract]
#6	Aphthous Ulcer [Title/ Abstract]
#7	Aphthous Ulcers [Title/ Abstract]
#8	Ulcers, Aphthous [Title/ Abstract]
#9	Aphthae [Title/ Abstract]
#10	Canker Sore [Title/ Abstract]
#11	Canker Sores [Title/ Abstract]
#12	Sore, Canker [Title/ Abstract]
#13	Sores, Canker [Title/ Abstract]
#14	Periadenitis Mucosa Necrotica Recurrens [Title/ Abstract]
#15	#1 OR #2 OR #3 OR #4 OR #5 OR #6 OR #7 OR #8 OR #9 OR #10 OR #11 OR #12 OR #13 OR #14
#16	Paeonia [MeSH Terms]
#17	Radix, Paeoniae [Title/ Abstract]
#18	Paeonias [Title/ Abstract]
#19	Peony [Title/ Abstract]
#20	Peonies [Title/ Abstract]
#21	Total glucosides of peony [Title/ Abstract]
#22	Total glucosides of paeonia [Title/ Abstract]
#23	Total glucosides of paeony [Title/ Abstract]
#24	TGP [Title/ Abstract]
#25	Peoniflorin [Title/ Abstract]
#26	Paeoniflorin [Title/ Abstract]
#27	Peoniflorin sulfonate [Title/ Abstract]
#28	Baishao [Title/ Abstract]
#29	#16 OR #17 # OR 18 OR #19 OR #20 OR #21 OR #22 OR #23 OR #24 OR #25 OR #26 OR #27 OR #28
#30	#15 AND #29
The search strategy for Embase	
Number	Search terms
#1	'Aphthous Stomatitis'/exp
#2	'Aphthous Stomatitides':ab,ti
#3	'Aphthous Ulcer':ab,ti
#4	'Aphthous Ulcers':ab,ti

#5	'Aphthae':ab,ti
#6	'Canker Sore':ab,ti
#7	'Canker Sores':ab,ti
#8	'Periadenitis Mucosa Necrotica Recurrens':ab,ti
#9	#1 OR #2 OR #3 OR #4 OR #5 OR #6 OR #7 OR #8
#10	'Paeonia'/exp
#11	'Paeoniaceae':ab,ti
#12	'Paeonias':ab,ti
#13	'Peony':ab,ti
#14	'Peonies':ab,ti
#15	'Total glucosides of peony':ab,ti
#16	'Total glucosides of paeonia':ab,ti
#17	'Total glucosides of paeony':ab,ti
#18	'TGP':ab,ti
#19	'Peoniflorin':ab,ti
#20	'Paeoniflorin':ab,ti
#21	'Peoniflorin sulfonate':ab,ti
#22	'Baishao':ab,ti
#23	#10 OR #11 OR #12 OR #13 OR #14 OR #15 OR #16 OR #17 OR #18 OR #19 OR #20 OR #21 OR #22
#24	#9 AND #23

The search strategy for Cochrane Library

Number	Search terms
#1	MeSH descriptor: [Stomatitis, Aphthous] explode all trees
#2	(Aphthous Stomatitides):ti,ab,kw
#3	(Aphthous Stomatitis):ti,ab,kw
#4	(Stomatitides, Aphthous):ti,ab,kw
#5	(Ulcer, Aphthous):ti,ab,kw
#6	(Aphthous Ulcer):ti,ab,kw
#7	(Aphthous Ulcers):ti,ab,kw
#8	(Ulcers, Aphthous):ti,ab,kw
#9	(Aphthae):ti,ab,kw
#10	(Canker Sore):ti,ab,kw
#11	(Canker Sores):ti,ab,kw
#12	(Sore, Canker):ti,ab,kw
#13	(Sores, Canker):ti,ab,kw
#14	(Periadenitis Mucosa Necrotica Recurrens):ti,ab,kw
#15	#2 OR #3 OR #4 OR #5 OR #6 OR #7 OR #8 OR #9 OR #10 OR #11 OR #12 OR #13 OR #14
#16	#1 OR #15
#17	MeSH descriptor: [Paeonia] explode all trees
#18	(Radix, Paeoniae):ti,ab,kw
#19	(Paeonias):ti,ab,kw

#20	(Peony):ti,ab,kw
#21	(Peonies):ti,ab,kw
#22	(Total glucosides of peony):ti,ab,kw
#23	(Total glucosides of paeonia):ti,ab,kw
#24	(Total glucosides of paeony):ti,ab,kw
#25	(TGP):ti,ab,kw
#26	(Peoniflorin):ti,ab,kw
#27	(Paeoniflorin):ti,ab,kw
#28	(Peoniflorin sulfonate):ti,ab,kw
#29	(Baishao):ti,ab,kw
#30	#18 OR #19 OR #20 OR #21 OR #22 OR #23 OR #24 OR #25 OR #26 OR #27 OR #28 OR #29
#31	#17 OR #30
#32	#16 AND #31

The search strategy for Web of science

#1	TS=("Stomatitis, Aphthous" OR "Aphthous Stomatitides" OR "Aphthous Stomatitis" OR "Stomatitides, Aphthous" OR "Ulcer, Aphthous" OR "Aphthous Ulcer" OR "Aphthous Ulcers" OR "Ulcers, Aphthous" OR "Aphthae" OR "Canker Sore" OR "Canker Sores" OR "Sore, Canker" OR "Sores, Canker" OR "Periadenitis Mucosa Necrotica Recurrens")
#2	TS=("Paeonia" OR "Radix, Paeoniae" OR "Paeonias" OR "Peony" OR "Peonies" OR "Total glucosides of peony" OR "Total glucosides of paeonia" OR "Total glucosides of paeony" OR "TGP" OR "Peoniflorin" OR "Paeoniflorin" OR "Peoniflorin sulfonate" OR "Baishao")
#3	#1 AND #2

The search strategy for CNKI

（SU='芍药' OR SU = '白芍' OR SU = '白芍总苷' OR SU = '白芍总苷胶囊' OR SU = '帕夫林') AND (SU ='复发性阿弗他溃疡' OR SU = '复发性口腔溃疡'）

The search strategy for WanFang Database

主题:("芍药" OR "白芍" OR "白芍总苷" OR "白芍总苷胶囊" OR "帕夫林") AND 主题:("复发性阿弗他溃疡" OR "复发性口腔溃疡")

**The search strategy for VIP Database**
((M=芍药 OR 白芍 OR 白芍总苷 OR 白芍总苷胶囊 OR 帕夫林) OR (K=芍药 OR 白芍 OR 白芍总苷 OR 白芍总苷胶囊 OR 帕夫林) OR (R=芍药 OR 白芍 OR 白芍总苷 OR 白芍总苷胶囊 OR 帕夫林)) AND ((M=复发性阿弗他溃疡 OR 复发性口腔溃疡) OR (K=复发性阿弗他溃疡 OR 复发性口腔溃疡) OR (R=复发性阿弗他溃疡 OR 复发性口腔溃疡))
**The search strategy for SinoMed**
("芍药"[摘要:智能] OR "白芍"[摘要:智能] OR "白芍总苷"[摘要:智能] OR "白芍总苷胶囊"[摘要:智能] OR "帕夫林"[摘要:智能]) AND("复发性阿弗他溃疡"[摘要:智能] OR "复发性口腔溃疡"[摘要:智能])

Supplementary Table S4. Efficacy evaluation criteria of overall response rate and significant response rate.

Author (publication year)	Efficacy evaluation criteria
Chen X. and Zhang H. L. (2021)	Significant response: The pain is significantly reduced, the ulcer is basically healed, and there is no recurrence in the 2-month follow-up.<br>Response: The pain is alleviated, the ulcer area has decreased, and there is no recurrence in a follow-up of 1 month.<br>Non-response: no significant change in ulcer area, pain, and recurrent episodes during follow-up.
Sun (2020)	IN grading
Yan and Zhang H. (2019)	Significant response: Three days after treatment, the pain disappeared and the ulcer healed completely.<br>Response: After 7 days of treatment, the pain disappeared and most of the ulcers healed.<br>Non-response: A situation in which the patient has not achieved effectiveness.
Yao et al. (2017)	Significant response: The healing time of ulcers was shortened significantly, the number of ulcers occurred was less, the interval was long, and the VAS was low.<br>Non-response: The healing time of ulcers, number of ulcers, interval, and VAS did not change significantly.<br>Response: All situations except the two mentioned above.
Xu and Chen Z. (2017)	Local efficacy evaluation criteria: Significant response: The average ulcer healing time was significantly shortened, and the VAS decreased; Response: The average ulcer healing time was shortened, or VAS decreased; Non-response: The average healing time of ulcer and VAS did not show significant changes.<br>Long-term efficacy evaluation criteria: IN grading
Su and Nong (2014)	Significant response: The healing time of ulcers was significantly shortened, and the VAS was significantly decreased.<br>Response: The healing time of ulcers was slightly shortened or VAS was slightly decreased.<br>Non-response: The healing time of ulcers and VAS score was not changed.
Wang et al. (2013)	IN grading
Tao (2012)	IN grading

IN grading: The efficacy evaluation criteria for treatment of RAU declared by Oral Mucosal Diseases Committee of Chinese Stomatological Association. Interval, I: the number of days without ulcers during the observation period. Evaluation method: I1: interval prolonged; I0: interval did not change or shorten. Number, N: the number of ulcers during a single bout of RAU. Evaluation method: N1: number of ulcers decreased; N0: number of ulcers did not change or increase. Efficacy determination: Significant response: I1N1, Response: I1N0 or I0N1, Non-response: I0N0. Overall response rate= response rate + significant response rate.

Supplementary Table S5. Changes of I^2 by omitting the included studies one by one.

Outcome	The study omitted	I^2
**VAS**		91%
	Su and Nong (2014)	92%
	Yao et al. (2017)	91%
	Yan and Zhang H. (2019)	92%
	Chen X. and Zhang H. L. (2021)	91%
	Liu Z. et al. (2023)	93%
	Liu Z. et al. (2023)	84%
**Overall response rate**		78%
	Tao (2012)	81%
	Wang et al. (2013)	22%
	Su and Nong (2014)	82%
	Yao et al. (2017)	81%
	Xu and Chen Z. (2017)	79%
	Xu and Chen Z. (2017)	79%
	Yan and Zhang H. (2019)	81%
	Sun (2020)	74%
	Chen X. and Zhang H. L. (2021)	81%
**Healing time**		94%
	Xu and Chen Z. (2017)	96%
	Yan and Zhang H. (2019)	0%
	Chen X. and Zhang H. L. (2021)	94%
**Interval**		97%
	Wang et al. (2013)	84%
	Xu and Chen Z. (2017)	0%
	Liu Z. et al. (2023)	99%

Supplementary Table S6. Reports on the number of ulcers.

Author（publish year）	Method of calculation	Observation period, weeks	Results		
			T	C	*p*-value
Liu Z. et al. (2023)	The sum of the number of oral ulcers per day in a month	0-4	35.4 (15.0, 51.4)	23.6 (11.8, 43.9)	0.17
		5-12	23.0 (13.9, 47.7)	21.4 (16.1, 31.1)	0.78
		13-24	16.0 (10.5, 32.2)	15.0 (4.8, 23.1)	0.24
		25-36	7.6 (4.2, 14.4)	28.2 (10.9, 45.7)	<0.001
Xu and Chen Z. (2017)	Total number of ulcer occurred during the observation period	More than 1 year	2.4±1.2	3.1±1.5	<0.05
Wang et al. (2013)	Total number of ulcer occurred during the observation period	0-4	4.54±1.29	7.66±2.68	<0.01
		5-8	4.00±1.12	7.30±2.80	<0.01
		9-12	3.48±1.23	6.28±2.93	<0.01
		13-16	3.06±0.82	5.76±2.33	<0.01
		17-20	3.24±1.17	5.20±1.87	<0.01
		21-24	3.02±1.13	5.26±2.05	<0.01

T, treatment group; C, control group. Data were present as mean ± standard deviation or median (interquartile spacing).

Supplementary Table S7. .

Authors (publication year)	Interventions		Detailed information of the drugs
	T	C	
Liu Z. et al. (2023)	TGP (0.6g tid), Kangfuxin liquid, and Tong Ren Tang Oral Ulcer Powder	Placebo, Kangfuxin liquid, and Tong Ren Tang Oral Ulcer Powder	TGP (trade name: Pavlin; produced by Ningbo Liwah Pharmaceutical Co., Ltd., H20055058, lot 191115, Paeonia lactiflora Pall. 0.3 g/capsule, containing 130 mg of paeoniflorin); Kangfuxin liquid (Kunming Sino Pharmaceutical Co., Ltd., GYZ53020054, 50 mL-2 bottles/box); Tong Ren Tang Oral Ulcer Powder (Tong Ren Tang Pharmaceutical Factory, Beijing Tong Ren Tang Co., Ltd., GYZ11020184, 3 g/bottle)
Chen X. and Zhang H. L. (2021)	TGP (0.6g bid), vitamin B, and thalidomide	Vitamin B and thalidomide	TGP (Ningbo Liwah Pharmaceutical Co., Ltd., H20055058, Paeonia lactiflora Pall. 0.3 g/capsule); Thalidomide tablets (Changzhou Pharmaceutical Co., Ltd., H32026129, 25 mg/tablet); Vitamin B (Guangdong Hengjian Pharmaceutical Co., Ltd., H44021164, 0.5 mg/tablet)
Sun (2020)	TGP (0.6g tid)	Vitamins with minerals tablets	TGP (Ningbo Liwah Pharmaceutical Co., Ltd., Paeonia lactiflora Pall. 0.3 g/capsule); Vitamins with minerals tablets (trade name: 21 Jin Weita Pian; Hangzhou Minsheng Health Pharmaceutical Co., Ltd.)
Yan and Zhang H. (2019)	TGP (0.6g tid)	Vitamin B, vitamin C, and zinc	TGP (Ningbo Liwah Pharmaceutical Co., Ltd., H20055058, Paeonia lactiflora Pall. 0.3 g/capsule); Vitamin B (Beijing Haidrun Pharmaceutical Group Co., Ltd., H42020613); Vitamin C (Huazhong Pharmaceutical Co., Ltd., H42020614); Zinc (Hainan Pharmaceutical Factory Co., Ltd., H46020030); Iodine glycerin (Beijing Haidrun Pharmaceutical Group Co., Ltd., H11021298)
Yao et al. (2017)	TGP (0.6g tid)	Vitamin B_2	No Drug Detailed Information Provided.
Xu and Chen Z. (2017)	TGP (0.6g bid, 5 times per week), thalidomide, vitamin B, vitamin E, vitamin A	Thalidomide, vitamin B, vitamin E, vitamin A	No Drug Detailed Information Provided.
Su and Nong (2014)	TGP (0.6g tid)	Vitamin B_2	TGP (Ningbo Liwah Pharmaceutical Co., Ltd., H20055058, Paeonia lactiflora Pall. 0.3 g/capsule); Vitamin B2 (Guangdong Hengjian Pharmaceutical Co., Ltd., H44020621)
Wang et al. (2013)	TGP (0.6g tid), Compound chlorhexidine, dexamethasone patching agent, and oral cleanser	Vitamin B_2, Compound chlorhexidine, dexamethasone patching agent, and oral cleanser	TGP (Ningbo Liwah Pharmaceutical Co., Ltd., Paeonia lactiflora Pall. 0.3 g/capsule); Vitamin B2 (Huazhong Pharmaceutical Co., Ltd., 5 mg/tablet); No detailed information provided for the other medications.

Tao (2012)	TGP (0.6g tid)	Vitamins with minerals tablets	TGP (trade name: Pavlin; Ningbo Liwah Pharmaceutical Co., Ltd.,); Vitamins with minerals tablets(trade name: Jinshikang)
